# Pulmonary Tuberculosis and Sarcoidosis Presented With Pleuropericardial Effusion: A Challenging Case Report

**DOI:** 10.1155/crdi/5770992

**Published:** 2026-07-26

**Authors:** Atousa Hakamifard, Sara Abolghasemi, Mitra Heidarpour, Mahnaz Momenzadeh

**Affiliations:** ^1^ Cancer Prevention Research Center, Isfahan University of Medical Sciences, Isfahan, Iran, mui.ac.ir; ^2^ Infectious Diseases and Tropical Medicine Research Center, Shahid Beheshti University of Medical Sciences, Tehran, Iran, sbmu.ac.ir; ^3^ Department of Pathology, School of Medicine, Isfahan University of Medical Sciences, Isfahan, Iran, mui.ac.ir; ^4^ Department of Clinical Pharmacy and Pharmacy Practice and Pharmaceutical Sciences Research Center, Isfahan University of Medical Sciences, Isfahan, Iran, mui.ac.ir

**Keywords:** case report, non-necrotizing granuloma, pleuropericardial effusions, sarcoidosis, tuberculosis

## Abstract

Differentiating tuberculosis (TB) from sarcoidosis, especially in TB endemic regions, is one of the major clinical challenges because these two diseases may show considerable similarity in terms of clinical manifestations, imaging findings, and granulomatous pattern. In addition, the presence of noncaseating granuloma alone is not sufficient for the definitive diagnosis of sarcoidosis because in some cases, TB may also show granulomas without necrosis. In this report, a 83‐year‐old man is presented with fever, productive cough, chest pain, weakness, and mild dyspnea, who had a history of pleural and pericardial effusion, with elevated pericardial adenosine deaminase (ADA), a positive tuberculin skin test, and pleural granuloma. The patient was initially treated with anti‐TB therapy for probable diagnosis of TB pleurisy and also corticosteroid for probable sarcoidosis, but because of the development of drug‐induced hepatitis, treatment was interrupted and modified several times. On subsequent presentation, chest high‐resolution computed tomography (HRCT) showed pericardial thickening, pericardial effusion, pulmonary nodules with a perilymphatic distribution, right hilar lymphadenopathy, and a cavitary lesion in the right upper lobe. Sputum smear was 3+ positive for acid‐fast bacilli, and anti‐TB treatment was reinitiated. Nevertheless, because of the imaging pattern, relative response to corticosteroid, and pathologic findings including non‐necrotizing granulomas, the possibility of concurrent sarcoidosis was also raised. After evaluating the possibility of TB progression and the effect of interruptions in anti‐TB therapy caused by drug‐induced hepatitis, the patient was treated with anti‐TB regimen together with prednisolone and later azathioprine. Hence, the clinical case of pulmonary TB and probable sarcoidosis has been described in this study. The patient underwent anti‐TB and immunosuppression therapy and experienced significant improvement.

## 1. Introduction

Sarcoidosis is a multisystem granulomatous inflammatory disease of unknown etiology. Although the lungs and intrathoracic lymph nodes are the most common sites of involvement, sarcoidosis should not be considered solely a pulmonary disease, as almost any organ may be involved. Extrapulmonary manifestations may involve the skin, eyes, heart, nervous system, liver, spleen, and musculoskeletal system. Importantly, the pattern of organ involvement is not the same in all patients and may vary according to age, sex, geographical region, and especially ethnic background. For example, multiorgan and extrapulmonary involvement has been reported more frequently in Black patients, whereas in the Japanese population, ocular and cardiac involvement appears to be relatively more prominent. Therefore, recognizing sarcoidosis as a systemic disease with diverse pulmonary and extrapulmonary manifestations is of great importance, particularly when differentiating it from tuberculosis (TB) [1–4].

In contrast, TB is an infectious disease caused by *Mycobacterium tuberculosis*, which according to current data most commonly affects the lungs although extrapulmonary manifestations are also possible [5].

Although caseating granulomas are classically considered as one of the characteristic pathological findings in TB, granulomas caused by TB are not always necrotic. In some patients with TB, granulomas may appear as non‐necrotic or noncaseating, and even in a single patient, different granuloma patterns may coexist simultaneously. Therefore, the observation of a noncaseating granuloma in a biopsy specimen alone is not specific to sarcoidosis and cannot rule out TB. Pathological findings should always be interpreted together with microbiological evidence, imaging findings, and the patient’s clinical context [6].

The principal challenge in differentiating sarcoidosis and TB arises from the fact that both diseases can present with fever, weight loss, fatigue, predominant thoracic involvement, lymphadenopathy, pulmonary nodules, and granulomatous inflammation. Consequently, their clinical, radiologic, and pathologic overlap especially in regions with high TB burden is considerable [7, 8]. The difficulty in distinguishing these diseases, therefore, lies not merely in their shared pulmonary involvement, which is common to both, but in their similar patterns of presentation despite fundamental differences in therapy. While TB requires antimycobacterial treatment, symptomatic or progressive sarcoidosis is usually managed with corticosteroids and other immunosuppressive agents; thus, misdiagnosis particularly erroneous initiation of immunosuppression in a TB patient may entail serious clinical consequences [1, 5, 7, 8].

Furthermore, some authors have proposed that TB and sarcoidosis may represent two distinct manifestations along a shared granulomatous spectrum driven by common immunoinfectious triggers [7]. In this context, the hypothesis by Dubaniewicz and colleagues is of special interest: they suggested that mycobacterial antigens, in hosts with different genetic backgrounds, may elicit divergent immune responses and ultimately result in distinct phenotypes of TB or sarcoidosis. They also indicated that, in some sarcoidosis patients—particularly before the initiation of corticosteroids or anti‐TNF‐*α* therapies—the consideration of immunogenetic background and even genotyping could be useful for assessing TB risk and moving toward personalized treatment [9].

Accordingly, the significance of the present report lies not merely in concurrent pulmonary involvement which is common to both diseases but in the coexistence of granulomatous processes and the need to integrate clinical, pathologic, and microbiologic evidence to guide simultaneous anti‐TB and anti‐inflammatory therapy. From this perspective, this case underscores the importance of a staged and multidisciplinary approach in patients suspected of overlapping TB and sarcoidosis [1, 2, 5, 8].

The present study reports a challenging case of microbiologically confirmed pulmonary TB accompanied by findings suggesting the possibility of concurrent sarcoidosis or a sarcoidosis‐like granulomatous reaction. The significance of this case is not solely due to pulmonary involvement since the lungs are commonly involved in both TB and sarcoidosis. The clinical importance of this case relates more to the considerable overlap of clinical, radiological, and pathological findings, the presence of pleuropericardial involvement, the repeated observation of non‐necrotic granulomas, and the need to strike a balance between anti‐TB treatment and the cautious use of anti‐inflammatory or immunosuppressive therapy.

Written informed consent was obtained from the patient for publication of this case report and the accompanying clinical data and images. All patient‐identifying information has been removed to preserve confidentiality.

## 2. Case Presentation

The patient is an 83‐year‐old man who was admitted to our hospital for the first time with fever, productive cough, chest pain, mild dyspnea, and weakness. About 3 months before this admission, the patient was evaluated in another hospital with a history of shortness of breath and chest pain, without cough or sputum. That time, he was afebrile, and in the findings of the lung CT scan, pericardial effusion and pleural effusion have been reported. The pleural fluid was exudative with lymphocytic predominance. Pleural fluid smear, culture, tuberculosis‐polymerase chain reaction (TB‐PCR), and cytology were all reported negative, whereas adenosine deaminase (ADA) of pericardial fluid and pleural fluid was 58 U/L and 8.3 U/L, respectively. The patient was a candidate for pericardial and pleural biopsy. Pericardial fibrosis was reported in the pathology report. In the pleural biopsy, a noncalcified granuloma was reported but no signs of malignancy were seen. According to the pleural granulomatous inflammation, positive tuberculin skin test, and the regional epidemiologic context of TB, the diagnosis of TB pleurisy was made and anti‐TB therapy was initiated during that prior hospitalization. The patient was Iranian who live in Isfahan city, and the medical record contained no documented history of close contact with an individual diagnosed with active TB prior to the initial diagnosis.

The initial anti‐TB regimen included isoniazid, rifampin, pyrazinamide, and ethambutol. After 10 days, this regimen was discontinued because of drug‐induced hepatitis. After improvement in liver function tests, anti‐TB therapy was restarted; however, recurrent hepatotoxicity led to subsequent interruptions and regimen modification.

Interruptions in anti‐TB therapy were primarily due to recurrent drug‐induced hepatitis rather than documented intentional nonadherence by the patient. After each discontinuation, the treatment regimen was re‐evaluated and restarted or modified according to liver function tests and clinical status.

After 4 weeks, the patient underwent high‐resolution CT (HRCT) because of dyspnea and pleuritic chest pain, which revealed pleural effusion. The plural biopsy was repeated, and the result was the same. Also, according to the atrial fibrillation (AF) rhythm, the patient underwent anticoagulant therapy. Prednisolone 15 mg daily had been started during the previous admission due to persistent pleural and pericardial inflammation, recurrent effusions, and pleural granulomatous pathology. At that time, sarcoidosis was also considered as a possible explanation. However, since TB had not been completely ruled out, corticosteroid therapy was prescribed cautiously in conjunction with anti‐TB treatment rather than as monotherapy.

After 25 days, because of the worsening of symptoms this time, the patient referred to our hospital for more evaluation. The anti‐TB regimen was again discontinued for 1 week prior to this hospital admission due to drug‐induced hepatitis.

At this admission, the vital signs were as follows: blood pressure of 110/80 mmHg, pulse rate of 94 beats/min, respiratory rate of 24 breath/min, and temperature of 38.2°C. The initial laboratory tests at admission were as follows: white blood cells of 11,800/mm^3^, polymorph nuclear of 80%, hemoglobin of 11 gr/dL, platelet of 3,49,000/mm^3^, erythrocyte sedimentation rate of 35 mm/hr, and C‐reactive protein of 40 mg/dL. Aspartate aminotransferase and alanine aminotransferase were 47 U/L and 52 U/L, respectively (normal range up to 45).

According to AF rhythm, warfarin continuation, digoxin, and verapamil initiation were recommended in cardiac consultation.

Calcium and liver function tests were assessed; no hypercalcemia was found, and abnormal liver enzymes were attributed to drug‐induced hepatitis rather than hepatic sarcoidosis. No peripheral lymphadenopathy, ocular symptoms, cutaneous lesions, or neurological manifestations were documented.

Regarding pulmonary parenchymal involvement, chest HRCT demonstrated multiple lung nodules with predominantly perilymphatic distribution, especially in the upper lobes. Some nodules appeared in clusters forming areas of parenchymal consolidation. Interlobular septal thickening and fissural nodularity were also noted. A 12‐mm enlargement of the right hilar lymph node and a 13‐mm cavitary lesion were observed in the right upper lobe. The radiology report suggested three possible differential diagnoses: sarcoidosis, TB, or malignancy (Figure 1).

**FIGURE 1:**
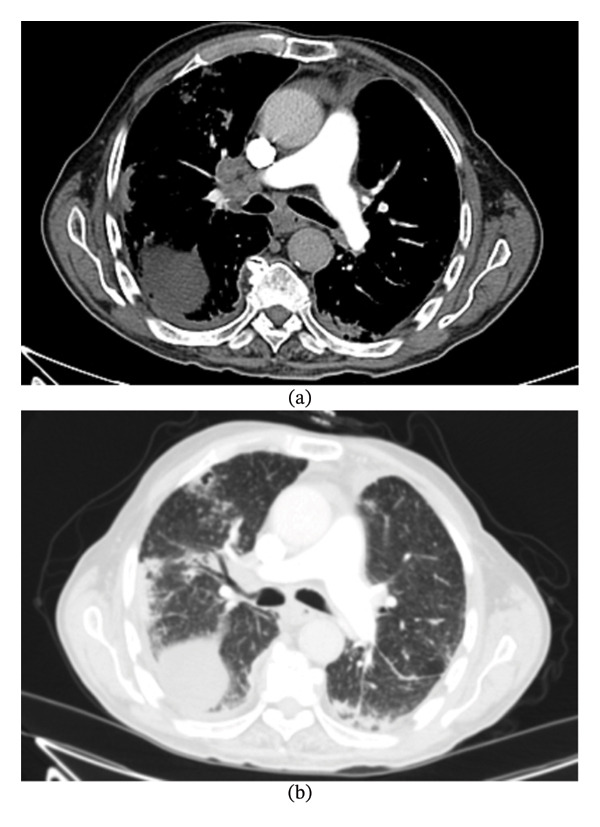
Thoracic CT scan: (a) lung window: parenchymal cavitary nodule and mass in RUL (short arrow) axial interstitial thickening and left major fissure nodularity (large arrow) are present. (b) Mediastinal window: right hilar and subcarinal discrete lymphadenopathy are seen.

Sputum study was conducted, and the results were positive for acid‐fast bacilli (AFB) 3+. An anti‐TB regimen was started for the patient again. Liver enzymes ALT increased to 245 U/L, AST to 378 U/L after 3 days of starting anti‐TB drugs, so the drugs were stopped because of hepatitis.

The rheumatology panel and serum angiotensin‐converting enzyme (ACE) level were in the normal range. Human immunodeficiency virus was negative. Based on the relative response to prednisolone, a lung CT‐guided biopsy was performed for better evaluation. The pathology result is shown in Figure 2.

**FIGURE 2:**
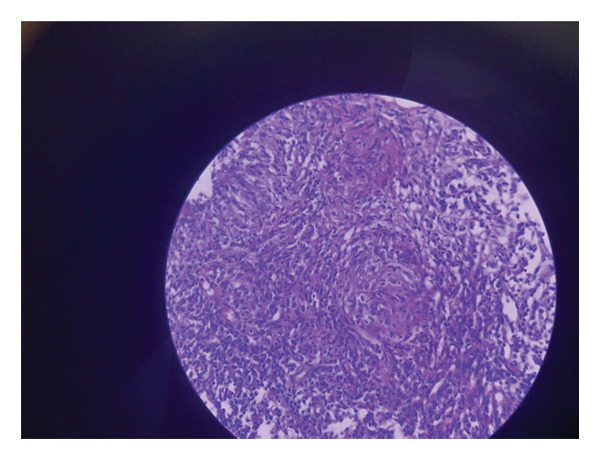
Non‐necrotizing granulomas (well defined borders, without central necrosis) x400.

Pulmonary TB was supported microbiologically by a positive AFB sputum smear. Associated imaging and pathologic findings raised the possibility of concurrent sarcoidosis; however, the definite coexistence of both diseases could not be confirmed with certainty.

Azathioprine was not started based on definitive evidence of sarcoidosis but was added later after re‐evaluation of the clinical course, HRCT findings, and repeated non‐necrotizing granulomas that raised the possibility of concurrent sarcoidosis. The patient was closely monitored for liver function, infectious complications, and clinical response. After 9 months of follow‐up, he remained well with no recurrence of symptoms. The timeline of diagnostic findings and the treatment course is shown in Table 1.

**TABLE 1:** Timeline of diagnostic findings and treatment course.

Time	Diagnostic findings	Treatment
Previous hospitalization, about 3 months earlier	Pleural and pericardial effusion, positive tuberculin skin test (TST), pleural granuloma	Initiation of antituberculosis regimen including isoniazid, rifampin, pyrazinamide, and ethambutol
10 days later	Drug‐induced hepatitis	Discontinuation of antituberculosis therapy
4 weeks later	Persistence of symptoms and effusion	Reinitiation of antituberculosis therapy with prednisolone 15 mg/day
Current hospitalization	Abnormal HRCT, sputum smear AFB 3+ positive	Reinitiation and modification of antituberculosis therapy
After re‐evaluation	Probability of sarcoidosis or sarcoid‐like reaction	Continuation of prednisolone and addition of azathioprine.
9‐month follow‐up	Clinical improvement	No recurrence of symptoms

## 3. Discussion

TB and sarcoidosis are diseases that have similar clinical characteristics, making it challenging to differentiate between the two, especially in endemic areas for TB. The management of these two is completely different; hence, establishing the diagnosis is crucial. In the literature, cases of concomitant TB and sarcoidosis are well described. Different hypotheses support that particular mycobacterial antigens could trigger immune responses, leading to sarcoidosis [10, 11]. Some data suggest that TB and sarcoidosis should be understood as different presentations along the spectrum of the same disease [12]. Also, a higher phagocytic activity of monocytes was described in both entities. However, an increased frequency of monocytes with FcγRIII (CD16) and decreased with CR1 (CD35) receptor in the blood of sarcoidosis was shown, which may account for the decreased clearance of immunocomplexes and different immune responses in the two diseases [13].

Our case highlights the questions about the relationship between sarcoidosis and TB, including TB predisposing the patient to sarcoidosis or sarcoidosis complicated with TB later due to the use of corticosteroids.

High awareness is critical in TB diagnosis, and laboratory diagnosis is based on several methods, such as direct microscopy, tissue culture, and PCR‐based nucleic acid amplification techniques. The sensitivity and specificity values with direct microscopy are 66% and 100%, respectively [14]. Culture continues to be the gold standard for diagnosing TB; however, isolation can take up to 6 weeks due to the slow growth rate of the organism [15]. Studies showed that PCR sensitivities ranged from 77% to more than 95%, and PCR specificities were > 95% for smear‐positive specimens [16, 17].

In 1960, Scadding described 230 cases and reported that the tubercle bacillus was found at some stage in 13% of the patients [18]. Since then, several studies have investigated the controversial relationship between sarcoidosis and TB.

Using PCR techniques, Saboor et al. [19] found MTB DNA in 50% of sarcoidosis lung samples and identified non‐MTB DNA in the other 20%. Wang et al. [20] found that patients with TB were 8.08 times more likely to develop sarcoidosis than non‐TB patients during 5 years of observation. On the other hand, in patients with sarcoidosis, the risk of TB was 1.85 times compared to nonsarcoidosis patients, and this risk was significant only in a 1‐year follow‐up period.

Diffuse miliary opacities pointed to millet grains are seen in both diseases but are rare in sarcoidosis. Bilateral hilar lymphadenopathy alone or with mediastinal lymphadenopathy is sarcoidosis’s most common radiographic manifestations. Parenchymal nodules in sarcoidosis are usually bilateral, symmetrical, and well defined in a perilymphatic distribution [20], while the diffusion pattern in TB is usually unilateral lymphadenopathy, cavitation, and miliary. Micronodules are randomly distributed throughout the lungs in miliary sarcoidosis or TB [21]. In histopathologic evaluation, TB is characterized by caseating granulomas, whereas noncaseating granulomas characterize sarcoidosis. When caseous necrosis is not seen, and acid‐fast staining is negative in the biopsy sample, differentiation between TB and sarcoidosis is difficult [22].

It must be emphasized that non‐necrotizing granulomas are not specific to sarcoidosis and may occur in TB. Previous studies have shown that a subset of TB patients may exhibit epithelioid or noncaseating granulomas lacking classical caseous necrosis. Therefore, the presence of a non‐necrotizing granuloma in a single biopsy specimen cannot alone confirm sarcoidosis or rule out TB.

The clinical improvement observed after addition of azathioprine should be interpreted cautiously. Since the patient was simultaneously receiving anti‐TB regimen, this improvement cannot be regarded as definitive evidence of sarcoidosis or a direct response to azathioprine. Part or even most of the clinical improvement may have resulted from effective continuation of anti‐TB treatment after previous interruptions due to drug‐induced hepatitis. Thus, the therapeutic response in this patient merely suggests possible concurrent sarcoidosis and does not confirm the definite coexistence of sarcoidosis and TB [23–25].

Initially, the administration of anti‐TB drugs for the patient was empirical before referral to our hospital, considering the presence of pleural granulomas in the patient in TB endemic areas, and it was completely logical. There are some hypotheses for this case according to his scenario: First, it is possible that paradoxical IRIS began with the initiation of anti‐TB treatment and symptoms continued to progress, so pulmonary TB also developed along with TB pleurisy and then the TB triggered occurrence of sarcoidosis. The second possibility may be that pleural granuloma was initially attributed to sarcoidosis instead of TB and then the initiation of corticosteroid in this old patient caused TB reactivation as pulmonary TB. In this scenario considering that the patient was on the anti‐TB regimen, the reactivation of TB could be justified by irregular use of anti‐TB drugs due to the frequent occurrence of drug‐induced hepatitis. Third, the patient may have been diagnosed with TB pleurisy and sarcoidosis simultaneously and then became progressive, which seems to be a rarer hypothesis. Forth, this case may be a TB case, with a possibility of sarcoid‐like feature, and the diseases progress because the anti‐TB might not sufficiently work due to drug‐induced hepatitis.

The positive AFB sputum smear strongly supported active pulmonary TB. In addition, repeated interruptions of anti‐TB therapy due to drug‐induced hepatitis may have reduced initial treatment efficacy and contributed to persistence or worsening of symptoms. Therefore, subsequent clinical improvement cannot be attributed solely to azathioprine or corticosteroids since the patient was simultaneously receiving a modified anti‐TB regimen.

## 4. Strengths

One of the main strengths of the present study is that it addresses an important and real challenge in clinical medicine, namely, the differentiation of active pulmonary TB from sarcoidosis or a sarcoid‐like reaction, especially in a region where TB remains epidemiologically relevant.

Another strength is the presentation of a complex case with simultaneous involvement of multiple systems, including the lungs, pleura, and pericardium. This feature makes the present report educationally valuable, as it shows that TB and sarcoidosis may have very similar clinical, radiologic, and pathologic manifestations.

Moreover, the current study highlights the importance of cautious interpretation of pathology findings. The presence of non‐necrotizing granuloma is usually considered in favor of sarcoidosis, but this report shows that such a finding alone is not sufficient to exclude tuberculosis or confirm sarcoidosis.

Another strength is the attention paid to the effect of drug‐induced hepatitis caused by anti‐TB medications on the patient’s treatment course. This issue is clinically very important because interruption and reinitiation of anti‐TB treatment can both affect the therapeutic response and make diagnostic interpretation more difficult.

Besides, this study emphasizes the importance of cautious decision‐making regarding immunosuppressive therapy. In a patient with evidence suggestive of active TB, the initiation of corticosteroids or azathioprine may be dangerous; therefore, the present report has high clinical value in demonstrating the necessity of close monitoring and concurrent anti‐TB treatment.

### 4.1. Limitations of the Study

This report has several diagnostic limitations. First, reliance on a single biopsy site showing non‐necrotizing granuloma reduces the certainty of diagnosing sarcoidosis because granulomatous lesions in TB may be heterogeneous and may contain both caseating and noncaseating granuloma. Second, since azathioprine was added concurrently with the continuation of anti‐TB treatment, the subsequent clinical improvement cannot be attributed solely to immunosuppressive therapy. For this reason, we interpret this case more cautiously as microbiologically confirmed pulmonary TB with the possibility of concurrent sarcoidosis. Third, repeated interruptions of anti‐TB therapy due to drug‐induced hepatitis are one of the important limitations in interpreting the patient’s clinical course. Although there was no documented evidence of intentional nonadherence to treatment by the patient, these treatment gaps may have reduced the effectiveness of anti‐TB therapy and complicated evaluation of the treatment response.

## 5. Conclusion

This case demonstrates the diagnostic and therapeutic challenge of distinguishing active pulmonary TB from possible concurrent sarcoidosis or sarcoid‐like granulomatous reaction. In patients with microbiologic evidence of TB, the presence of non‐necrotizing granuloma, lymphadenopathy, or partial response to corticosteroids should not be regarded as definitive evidence of sarcoidosis. The therapeutic response should be interpreted cautiously, especially when anti‐TB therapy has been interrupted and subsequently modified due to drug‐induced hepatitis. Precise microbiologic assessment, clear delineation of the treatment timeline, and close monitoring before and during immunosuppressive therapy are essential.

## Author Contributions

Atousa Hakamifard contributed in case selection and study design. Mahnaz Momenzadeh, Sara Abolghasemi, and Mitra Heidarpour contributed in database searches and extraction of clinical studies and patient’s paraclinical information.

## Funding

No funding was obtained for this study.

## Disclosure

All authors wrote the original draft and have read and approved the final version of the manuscript.

## Consent

Informed consent was obtained from the participant.

## Conflicts of Interest

The authors declare no conflicts of interest.

## Data Availability

The data that support the findings of this case report are available from the corresponding author upon reasonable request.
